# A review of applications and limitations of using aquatic macroinvertebrate predators for biocontrol of the African malaria mosquito, *Anopheles gambiae* sensu lato

**DOI:** 10.1186/s13071-024-06332-3

**Published:** 2024-06-12

**Authors:** Hudson Onen, Martha A. Kaddumukasa, Jonathan K. Kayondo, Anne M. Akol, Frédéric Tripet

**Affiliations:** 1 Department of Zoology, Entomology and Fisheries Sciences, College of Natural Sciences, School of Biosciences, Makerere University, P.O Box 7062, Kampala, Uganda; 2 Department of Entomology, Uganda Virus Research Institute (UVRI), P.O Box 49, Entebbe, Uganda; 3 Swiss Tropical and Public Health Institute, Basel, Switzerland; 4 University of Basel, Basel, Switzerland; 5 Department of Biological Sciences, Faculty of Science, Kyambogo University, P.O. Box 1, Kampala, Uganda

**Keywords:** Biocontrol, Gut content, Macroinvertebrates, Malaria vectors, Predation experiment

## Abstract

Macroinvertebrate predators such as backswimmers (Heteroptera: Notonectidae), dragonflies (Odonata: Aeshnidae), and predatory diving beetles (Coleoptera: Dytiscidae) naturally inhabit aquatic ecosystems. Some aquatic ecosystems inhabited by these macroinvertebrate predator taxa equally form malaria vector larval habitats. The presence of these predators in malaria vector larval habitats can negatively impact on development, adult body size, fecundity, and longevity of the malaria vectors, which form important determinants of their fitness and future vectorial capacity. These potential negative impacts caused by aquatic macroinvertebrate predators on malaria vectors warrant their consideration as biocontrol agents in an integrated program to combat malaria. However, the use of these macroinvertebrate predators in malaria biocontrol is currently constrained by technical bottlenecks linked to their generalist predatory tendencies and often long life cycles, demanding complex rearing systems. We reviewed the literature on the use of aquatic macroinvertebrate predators for biocontrol of malaria vectors from the *An. gambiae* s.l. complex. The available information from laboratory and semi-field studies has shown that aquatic macroinvertebrates have the potential to consume large numbers of mosquito larvae and could thus offer an additional approaches in integrated malaria vector management strategies. The growing number of semi-field structures available in East and West Africa provides an opportunity to conduct ecological experimental studies to reconsider the potential of using aquatic macroinvertebrate predators as a biocontrol tool. To achieve a more sustainable approach to controlling malaria vector populations, additional, non-chemical interventions could provide a more sustainable approach, in comparison with the failing chemical control tools, and should be urgently considered for integration with the current mosquito vector control campaigns.

**Graphical Abstract:**

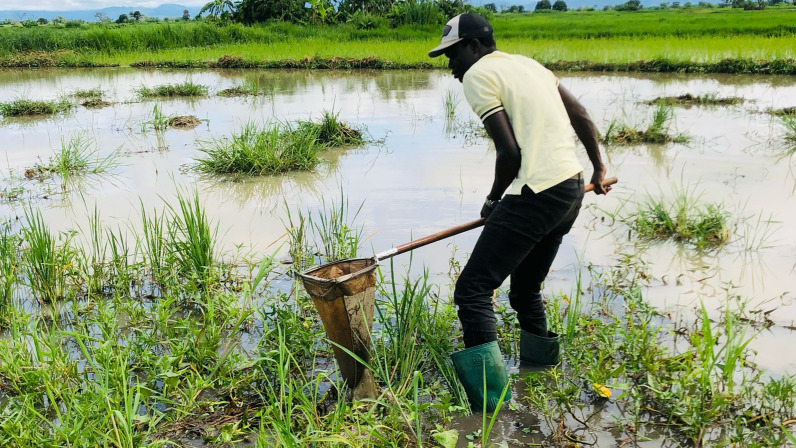

## Background

Macroinvertebrate predators naturally inhabit aquatic ecosystems that form larval breeding habitats of malaria vector species of the *Anopheles gambiae* complex (*An*. *arabiensis*, *An. gambiae* s. s., *An. coluzzii*, *An. merus*, and *An. melas*) [1, 2]. The abundance and composition of macroinvertebrate predator taxa supported by aquatic habitats of the *An. gambiae* s.l. species complex depends greatly on habitat permanency [3]. In West Africa, the more permanent larval breeding sites preferred by *An. coluzzii*, for example, support a greater number and diversity of macroinvertebrate predators than the temporary habitats of *An. gambiae* s.s. and *An. arabiensis* [4]. In East Africa, *An. gambiae* s.s. preferentially breeds in semi-permanent and ephemeral habitats compared with larger and permanent habitats such as ponds and streams that support a high diversity of macroinvertebrate predator taxa [5].

The presence of aquatic macroinvertebrate predators in larval habitats is known to influence the life-history traits of *An. gambiae* s.l., specifically larval development, adult body size, fecundity, and longevity, all of which can affect fitness and vectorial capacity [4, 6–9]. Furthermore, the selection pressures associated with predation by aquatic invertebrates have resulted in important adaptive avoidance mechanisms. For example, *An. gambiae* s.s. females have been shown to avoid laying eggs in water conditioned with backswimmers, *Notonecta* sp. This may suggest that kairomones associated with predators are shed in water that deters female mosquitoes from oviposition sites [6, 10]

Since the introduction of dichlorodiphenyltrichloroethane (DDT) in the 1940s, malaria prevention programs have relied heavily on chemical control approaches. Indoor residual spraying (IRS) of chemicals and insecticide-impregnated bed nets (ITNs) have been the preferred tools for targeting adult vectors indoors [11–13]. However, the effectiveness of these intervention is continuously being hampered by the rapid emergence and spread of resistance to commonly used insecticides in mosquito populations [14, 15]. Additionally, the selection pressures associated with indoor chemical control measures have led to an increase in outdoor biting behavior by some anopheline mosquito species, and changes in vector species dynamics [16, 17]. This outdoor biting behavior of some anopheline species aids residual malaria transmission.

The spread of insecticide resistance in mosquito populations is further thought to be fueled by agricultural practices. For example, the increased demand for food associated with Africa’s growing population has resulted in large areas of intensive rice cultivation coupled with heavy chemical pesticide application [18, 19]. The expansion of rice cultivations has also created large numbers of mosquito larval habitats often near human dwellings. Agricultural chemical pesticides contribute to a rise in insecticide resistance among malaria vectors because of the prolonged exposure of their immature stages to chemicals leaked into the aquatic larval habitats [20–23]. These low-specific agricultural chemicals can negatively affect natural aquatic mosquito predators even when they are directly applied as larvicides [24–27]. For example, common larvicides such as insect growth regulators (IGRs) and surface films (SFs) were found to be lethal to *Laccophilus* adults (Coleoptera: Dytiscidae) and dragonfly nymph at recommended concentrations [28].

Because of those significant challenges there is an urgent need for a shift in focus from chemical-based vector control approaches to integrated approaches tailored specifically for local settings. Such approaches can also take advantage of the local community knowledge, which encourages them to mobilize and directly get involved in malaria vector control efforts [29, 30]. Thus, there is a renewed interest in larval source management that involves community-based application of bio-larvicides [31, 32]. Ecologically friendly control approaches that employ aquatic macroinvertebrate predators could play a role in community-based vector control [33, 34]. Using aquatic macroinvertebrate predators would be advantageous because they are a natural component of the aquatic ecosystem where immature stages of the malaria vector breed [35]. This makes them potentially more accessible and affordable to rural communities than the expensive ITNs and IRS. Combining aquatic macroinvertebrate predators with other vector control methods could significantly contribute to a more sustainable long-term approach to reducing malaria vector populations. This is possible because all female mosquitoes, regardless of their biting behavior or insecticide resistance profile, lay eggs in aquatic larval habitats where macroinvertebrate predators potentially breed.

Despite these possible advantages that aquatic macroinvertebrate predators can offer in malaria vector control, no attempts have been made in the past to use them for malaria vector control, and they are currently rarely used as a biocontrol tool in malaria-endemic countries of Africa. This could be attributed to the complexities of their life cycle and their generalist tendencies [36–38], which make mass-rearing and large-scale deployment difficult. Thus, reassessing the possible efficacy of aquatic macroinvertebrate predators as biocontrol tools for malaria control and understanding the limitations preventing their broader use is critical.

Here, we review the available information on aquatic macroinvertebrate predators associated with *An. gambiae* s.l. larvae habitats and an explain the techniques available to study their preferences. Next, we evaluate their potential use as biocontrol agents, as well as limitations and prospects for successful applications. This information is critical to identifying the most important knowledge gaps in our understanding of the applied ecology of aquatic macroinvertebrate predators and in applied research required to enable new approaches to using aquatic predators for long-term malaria vector control.

### Techniques for identifying *An. gambiae* s.l. larval predators

In sub-Saharan Africa, immature stages of *An. gambiae* s.l. breed in various aquatic habitats ranging from small temporary rain pools to larger water pools such as rice fields [36, 37, 39, 40]. These habitats contain Planarians, Cladocerans, Copepods, and diverse assemblages of aquatic insect predators [5, 41–44]. The role that aquatic macroinvertebrate predators could play as biocontrol agents against *An. gambiae* s.l. is fairly established [45–47]. However, identifying the best predators for effective biocontrol against malaria vectors is not a straightforward task. Studies aimed at demonstrating predation of aquatic predators on given prey species generally fall into three main categories:

The first category is survey of aquatic habitats, describing patterns of co-occurrence or co-abundance of *An. gambiae* s.l. larvae and predatory taxa (Table 1). In their most basic form, such surveys are simply lists of taxa found in aquatic larval habitats [3, 4]. For example, a field survey in Tanzania has shown that order Hemiptera, Odonata, and Coleoptera were among the most abundant macroinvertebrate predators in the different malaria vector larval habitats along the Mara river [3]. These surveys, in their more elaborate form, may be classified by different types of larval habitats and correlate the presence or abundance of *An. gambiae* s.l. larvae with that of predator taxa within a habitat or different microhabitats, sometimes using clustering correlational approaches [5]. The main limitation of sampling surveys is that they infer potential predation on the basis of patterns of co-occurrence or abundance. This is correlative evidence that is best supported by studies that directly demonstrate prey–predation interactions [48, 49].

**Table 1:** Macroinvertebrate predators co-existing with *An. gambiae* s.l. in different aquatic habitat types in some malaria-prone countries as inferred through habitat sampling surveys or through predator gut contents analyses

Predator taxa^†^	Mosquito species	Habitat type(s)	Country	References*
Dragonflies (Libelluidae: Odonata), mayflies *Caenis macrura* (Caenidae: Ephemeroptera)*,* diving beetles (Dytiscidae: Coleoptera)	*An. pharoensis* and* An. sergentii*	Fresh water with loges and rocks. fallow fields with stagnant water	El-Galaa, El Nazlah, Youssef Al Seddik, and Tamiyyah district, Egypt	^1^[50]
Aeshinidae, Belostomatidae, Corixidae, Dytiscidae, Gomphidae, Libellulidae and Notonectidae	*An. + gambiae* s.l	Temporary natural habitats, wetlands, and ponds	Southwest Ethiopia	^1^[51]
Dytiscidae, water spiders (Cybaeidae: Arachnida)	*An. gambiae* s.l	Temporary pools	Villages of Kibuye and Kayonjo, Uganda	^1^[5]
Mayflies (Polymitarcyidae and Teloganodidae: Ephemeroptera), caddisflies (Hydropsychidae:Trichoptera), damselflies (Coenagrionidae: Zygoptera)	*An. arabiensis*	Semi-permanent and permanent water bodies fed by rainfall, floods, or both	Tubu village Okavango Delta, Botswana	^1^[52]
Hemiptera, Odonata, and Coleoptera	*An. gambiae* s.l., *An. coustani* complex, *An. maculipalpis*, *An. phaorensis*, *An. funestus* group, *An. azaniae*, *An. hamoni*, *An. christyi*, *An. ardensis*, *An. faini*, and *An. sergentii*	Rivers, drying streams, swamps, open puddles, rock pools, dam sites, hoof prints, vegetated pools, and drainage habitats	Mara river of Kenya and Tanzania	^1^[3]
Mayflies (Caenidae: Ephemeroptera), giant water bugs (Belostomatidae: Hemiptera), water boatmen (Corixidae: Hemiptera), toad bugs (Gelastochoridae: Heteroptera), water striders (Gerridae: Hemiptera), marsh treaders (Hydrometridae: Heteroptera), water treaders (Mesoveliidae: Hemiptera), creeping water bugs (Naucoridae: Heteroptera), water scorpions (Nepidae: Hemiptera), backswimmers (Notonectidae: Hemiptera), shore bugs (Saldidae: Hemiptera), broad-shouldered water striders (Veliidae: Hemiptera), predaceous diving beetles (Dytiscidae: Coleoptera), water scavenger beetles (Hydrophilidae: Coleoptera), water pennies (Psephenidae: Coleoptera), and marsh beetles (Scirtidae: Coleoptera)	*An. gambiae* s.l. and *An. coustani*	Burrow pits	Waktola, Ethiopia	^1^[53]
Chydoridae, Hydrachinidae, Psychodidae, Corixidae, Hydrometridae, Psycodidae, and Hydrachnidiae	*An. gambiae* s.l	Un-cemented pits, water puddles, ditches, lakeshore pools and swamps	Rusinga Island, Western Kenya	^1^[54]
Backswimmers, *Anisops jaczewskii* (Notonectidae: Hemiptera)	*An. gambiae* s.l	Irrigated rice field, rice puddles, temporary, rain-filled puddles, and quarries	Bama and Soumousso villages, Burkina Faso	^1^[55]
Backswimmers, *Anisops Anisops jaczewskii*, (Notonectidae: Hemiptera)	*An. gambiae* s.s. and *An. arabiensis*	Rice field irrigation canal	Bama village, Burkina Faso	^1^[42]
Water bugs *Diplonychus annulatus* (Belostomatidae: Hemiptera), *Diplonychus rusticus* (Belostomatidae: Hemiptera), *Ranatra* sp. water scorpions (Nepidae: Hemiptera), and water boatman *Sigara* sp. (Corixidae: Hemiptera)	*An. subpictus* and *An. culicifacies*	Ponds, rice fields, sewage drains, discarded plastic containers, and earthen pots	India	^1^[4]
Backswimmer, *Anisops* sp. and *Anithares* sp. (Notonectidae: Hemiptera), water boatman, *Micronecta* sp. (Corixidae: Hemiptera), dragonfly *Tramea* sp. (Libellulidae: Odonata), water scavenger beetles *Berosus* sp. (Hydrophilidae: Coleoptera), and diving beetles *Laccophilus* sp. (Dytiscidae: Coleoptera)	*An. gambiae* s.l	Rice fields	Bobo-Dioulasso, Burkina Faso	^1^[41]
Water scavenger beetles (Hydrophilidae: Coleoptera), predaceous diving beetles (Dytiscidae: Coleoptera), water boatman (Corixidae: Hemiptera), water scorpions (Nepidae: Hemiptera), giant water bugs (Belostomatidae: Hemiptera), green-eyed skimmers/emerald dragonflies (Cordulidae: Odonata) and backswimmers (Notonectidae: Hemiptera)	*An. gambiae* s.l	Drainage ditches, abandoned goldmines, and cow hoof prints	Western Kenya highlands	^1^[56]
Predaceous diving beetle, *Laccophilus* sp. (Dytiscidae: Coleoptera), water scavenger beetle, *Sternolophus* sp. and *Helochares* spp. (Hydrophilidae: Coleoptera), water boatman, *Micronecta scutellaris* (Corixidae: Hemiptera), and water scorpion, *Laccotrephes* (Nepidae: Hemiptera)	*An. gambiae* s.l.	Rice fields and temporary larval habitats	Kenya	^2^[57]
Wolf spiders (Lycosidae: Arachnida), Ephydridae, Dolichopodidae, Muscidae, Dytiscidae, Hydrophilidae, and Corixidae	*An. gambiae* s.l.	Small pools, ditches, and larger habitats	Kisumu, Kenya	^2^[58]
Tadpole shrimp, *Triops granarius* (Triopsidae: Notostraca)	*An. gambiae* s.l.	Temporary pools	Around huts in a Somali village	^1^[59]

^**†**^*In some studies, common, family, and order names were not provided by the authors*

^*^Citations refer to studies fitting the two categories discussed, namely

^*1*^*field sample surveys*

^*2*^*surveys with gut content analyses*

Surveys of aquatic macroinvertebrate predators’ gut contents can provide important confirmatory information on predator–prey preferences in their complex natural habitats. Different techniques, such as precipitin assays, polymerase chain reaction (PCR), enzyme electrophoresis, and immunological approaches, may be used [58, 60–62]. Other techniques using monoclonal and polyclonal antibodies to detect protein epitopes that allow for species-level identification of prey have also been proposed [63].

Such approaches have enabled the gaining of insights into the prey preferences of many aquatic macroinvertebrate taxa. Notably, electrophoretic analyses of the gut contents of backswimmers *Notonecta glauca* and *N. virzdis* (Notonectidae:Hemiptera) collected from Midleton, Ireland, revealed that these predators consumed *An. gambiae* s.l. as part of their natural diet [64]. Precipitin analysis of potential aquatic macroinvertebrate predators collected in Western Kenya revealed that immature *Pardosops* sp. and *Lycosa* sp. spiders (Lycosidae) preyed on *An. gambiae* s.l. [58]. The same approach was used in an extensive study of the gut contents of 2295 aquatic insect predators collected from rice fields, and 454 from temporary pools and ponds from Kenya. The study revealed that Coleoptera, Hemiptera, and Diptera were the most important *An. gambiae* s.l. larvae predators [57]. These analyses have limitations because aquatic macroinvertebrate predators that metabolize the ingested *An. gambiae* s.l. larvae quickly enough can be assigned false negatives [65]. This most likely contributed to the failure to identify some prey from the guts of previously studied aquatic macroinvertebrate predators [64]. As a result, a more sensitive, species-specific, and less expensive technology that can detect ingested prey several hours after predator ingestion is needed.

More recently, progress in molecular biology has enabled the use of polymerase chain reaction (PCR) for predation studies, which resulted in improved detection thresholds. For instance, an optimized PCR technique is shown to detect *An. gambiae* s.l. ingested by damselflies (Lestidae) and dragonflies (Libellulidae) after 1–6 h of ingestion [62, 65]. In Mbita, Western Kenya, PCR analyses of the guts of 330 aquatic insect predators revealed that dragonflies and damselflies (Odonata) had the highest (70.2%) positive rate of *An. gambiae* s.l., followed by water boatman bugs (Hemiptera) (62.8%) and beetles (Coleoptera) (18%) [66]. Interestingly, the results of analyses based on a ribosomal deoxyribonucleic acid polymerase chain reaction (rDNA-PCR) species diagnostic assay suggest that *An. gambiae* s.l.’s fourth-instar larvae can cannibalize conspecific first-instar larvae [67].

The decreasing costs of genomic approaches imply that DNA barcoding may now be used for comprehensive gut content analyses. This technique has been used to unravel the diet of field-collected *An. gambiae* s.l. larvae in Western Kenya [68]. Genomic techniques have been used successfully to reveal complex predator–prey interactions in spiders to understand the above- and below-ground food–web dynamics [69]. Therefore, while at the time of this review there was little evidence of barcoding analyses conducted on macroinvertebrate predators of mosquito larvae, prospects suggest that this tool will further facilitate gut content analysis studies. Gut content analyses are a powerful approach for demonstrating predatory interactions, however, they are dependent on sample sizes and power, the simultaneous presence of predators and mosquito prey in the sampled study area, and the rate of DNA degradation inside the predator’s gut. Although the gut content analyses approach is useful for incriminating the most common predators, such genomic studies are still comparatively costly, which may prevent the extensive longitudinal and horizontal sampling needed to fully unravel prey–predator networks across the different types of *An. gambiae* s.l. larval habitats.

Finally, predation experiments can establish whether a predatory taxon, previously identified through habitat and/or gut surveys, can effectively prey on *An. gambiae* s.l. larvae. These studies can also usefully complement gut content analyses by comparing different predator types and their consumption rates, or they may establish a relationship between prey size and their consumption rates [5, 51, 70, 71]. For example, semi-field experiments conducted in the Western Kenya highlands demonstrated that backswimmers (Notonectidae) were more effective predators of *An. gambiae* s.s. third-instar larvae and pupae than dragonfly nymphs (Libellulidae) or water scorpions (Belestomatidae) [45]. In semi-field studies in coastal Kenya, comparisons of predation rates on *Anopheles* larvae by five sympatric predatory taxa again showed that backswimmers consumed the most mosquito larvae, followed by water measurers/mash treaders (Hydrometridae), water striders (Gerridae), broad-shouldered water striders (Veliidae), and diving water beetles (Dysticidae) [72]. Whilst valuable, the results of experimental predation experiments may not reflect predation rates in the natural setting, where prey consumption may be constrained by availability and a variety of other abiotic and biotic factors.

### Potential aquatic macroinvertebrate predators for use as a biocontrol agents

A few insect taxa have emerged as prime candidates for use in biocontrol on the basis of evidence from the various approaches for identifying the most important predators of the African malaria mosquito described above. In sequential order of their importance, these are Odonata, Coleoptera, Hemiptera, and Diptera aquatic insects (Table 1).

Odonata is among the most effective aquatic macroinvertebrate predators of *An. gambiae* s.l. larvae. For example, during 1973 field surveys in Kenya, a precipitin test on the gut content of potential aquatic macroinvertebrate predators sampled from small pools and ditches revealed that dragonflies and damselflies nymphs preyed on *An. gambiae* s.l. larvae [58]. Conversely, five out of the nine Odonata species, *Agriocncmis inversa*, *Crocothemis etythraea*, *Pantala flavescens*, *Ischnura senegatensis*, and *Brachythemis lacustris*, sampled from the same location, tested positive for *An. gambiae* s.l., with *Ischnura senegatensis* dominating with 47.7% of positives [57]. The biocontrol efficacy of dragonfly nymphs *Brachytron pratense* against mosquito larvae *Anopheles subpictus* was demonstrated through a predation experiment conducted using 3 L water containers. The dragonfly *Brachytron pratense* nymphs consumed an average of 66 *An. subpictus’* fourth-instar larvae during a 24-h study period [73]. When the experiment was conducted under field conditions in the deeper 300L concrete water tanks, a significant decrease in the *An. subpictus* larval density was observed 15 days after the introduction of ten *B. pratense* nymphs [73]. The nymphs’ impact was further demonstrated, as the density of mosquito larvae significantly rebounded upon their removal [73]. In a PCR-based study focusing on gut contents of 330 aquatic macroinvertebrate predators sampled from six wetlands near Lake Victoria in Mbita, Western Kenya, 54.2% of the samples were found positive for *An. gambiae* s.l., and the Odonata had the highest average rate compared with other predatory orders with 70.2% positives [66].

Coleoptera also comprises important families of aquatic macroinvertebrate mosquito larvae predators [74]. Field surveys and gut content analysis demonstrated that some Coleoptera families, including the common Dytiscidae, co-exist and prey on *An. gambiae* s.l. (Table 1). Interestingly, adult diving beetles can fly from one aquatic habitat to another, and both adult and immature stages feed on mosquito larvae, thus making them particularly attractive predators [37, 74, 75].

The order Hemiptera contains taxa such as backswimmers (Notonectidae) that are also considered highly efficient predators of *An. gambiae* s.l. larvae [51]. Indeed, a semi-field study in Western Kenya showed that the backswimmer species, *Anisops debilis* (Notonectidae), is a more effective predator of *An. gambiae* s.l. larvae than other locally available Hemiptera predator species such as *Micrivelia* sp. (Veliidae), *Hydrometra* sp. (Hydrometridae), *Gerris hypolence* (Gerridae), and predacious diving beetle *Hydrovatus cribratus* (Dytiscidae) [72]. In a laboratory study conducted at Jimma University using aquatic macroinvertebrate predators collected from the Gilgel Gibe watershed, southwest Ethiopia, backswimmer (Notonectidae) was the most aggressive predator, with 71.5% daily mean predation on *An. gambiae* s.l. larvae compared with Dytiscidae, 67% [51]. In a separate semi-field study using field-collected aquatic macroinvertebrates from the same location [51], 89% of the belostomatids (*Hydrocyrius*) consumed *An. arabiensis* larvae placed in the artificial habitat compared with 64% of notonectids (*Notonecta, Anisops* and *Enithares*) and 56% of corixids (*Agraptocorixa*, *Micronecta*, *Sigara*, and *Trichocorixa*), respectively [70].

Taxa such as the dipterans also comprise promising candidate biocontrol agents. Mantis shore fly species, such as *Ochthera brevitibialis* and *chalybescens*, have been shown to prey on anopheline mosquito larvae and can reduce their populations locally [76, 77]. In deeper water, these species can easily catch anopheline larvae, whereas culicines can easily escape [76]. In predation experiments, the predatory shore fly *Ochthera chalybescens* preys on all stages of *An. gambiae* s.l., except for eggs [77]. In contrast to backswimmers, the efficiency of shore fly predation on *An. gambiae* s.l. was not affected by water surface/volume [51].

Cannibalism between mosquito species is also possible. Aside from the well-known mosquito taxa, *Culex toxorhynchites* species and *Culex tigripes*, whose larvae preferentially prey on mosquito larvae [78–80], there are scenarios where larvae of other mosquito taxa feeds on the dead larvae of other taxa. For example, when the third-instar *An. gambiae* s.s. larvae were placed in the same artificial habitat as the first-instar *Cx. quinquefasciatus* larvae and left to interact for some time, DNA of *Cx. quinquefasciatus* was detected in the *An. gambiae* s.s. gut content and vice versa [81]. A small number of dead first-instars were discovered in the controls, implying that some larvae in the treatment group were consumed after they died [81]. These findings imply that intraguild predation between the two species is common and that it is a voluntary process that is not triggered by food scarcity [81]. Interestingly, there is evidence that *An. gambiae* s.l. engages in intraspecies cannibalism. In laboratory experiments, the fourth-instar *An. gambiae* s.l. larvae were video-recorded cannibalizing eggs and large numbers of newly hatched first-instars [82].

Apart from aquatic insects, several spider families (Tetragnathidae, Lycosidae, Pisauridae, and Trechaleidae) prey on *An. gambiae* s.l. larvae in and around aquatic habitats [83]. To catch their prey, predatory spiders employ a variety of strategies. Those that prey on mosquito larvae, for example, are active hunters who do not build webs. These spiders are typically semi-aquatic, surface film locomotors, or deep divers [43]. Notably, semi-aquatic surface film locomotors such as *Dolomedes triton* (Pisauridae, Araneae) are active mosquito larvae predators [84]. Another active anopheline mosquito predator is *Argyroneta aquatica*, which hides inside a bell-shaped nest made of silk and submerged aquatic plants [85]. This spider species prefers hunting *Anopheles* over *Culex* larvae, regardless of the body size differences [85]. Although the intensity of spider predation may be low, a laboratory experiment has shown that a family such as Lycosidae consumes more than 84% of mosquito larvae in an artificial habitat of 2 × 1 × 4 m^3^ [86].

Other aquatic macroinvertebrate predators with biocontrol potential against *An. gambiae* s.l. larvae include crustaceans and planarians. For instance, Copepod, *Mesocyclops* sp. and *An. albimanus* mosquito larvae have been found to have a strong negative association in many ponds and small water bodies [87, 88]. Additionally, field trials in temporary pools, marshes, and rice fields have shown that copepods, *Mesocyclops*, can eliminate mosquito larvae, including *An. gambiae* s.l., provided the right species is introduced to the right habitat at the right time [89]. Copepods, like all aquatic predators, are generalists, which limits their effectiveness as a potential biocontrol tool. *Mesocyclops thermocyclopoides*, for example, prey on *An*. *stephensi* but prefer to feed on cladocerans when both are available [90].

Among the planarians, mesostoma is the most widely distributed and important mosquito larvae predator in small water bodies [91, 92]. Laboratory observations of some *Mesostoma* spp. collected from shallow aquatic habitats have revealed a wide variety of prey-killing mechanisms including mucus trapping, sit-and-wait predation, toxin release into water, and active searching [93, 94]. *Mesostoma* spp. was found to significantly reduce mosquito populations in plastic bowls during field experiments in residential areas [92]. During the dry season, the mosquito population was reduced to a mean of 352 (range 67–527) in the plastic bowls in 151 days, while the control group had only 93 (range 2–21) at the end of the same period. The feeding rate of 100 adult *Mesostoma* spp. on fourth-instar mosquito larvae is 10–12 mosquitoes per day, with the highest rate being 14 larvae per day [92]. The Asian planaria *Dugesia bengalensis* (Tricladida: Dugesiidae) is thought to be an effective predator of *An. gambiae* s.l. larvae. In a laboratory study, two batches of each of three large Petri dishes measuring 6 × 1 m^3^ were used, each containing 5–7 days starved matured planarians, and 50 *Anopheles* mosquito eggs, larvae, and pupae were exposed to the planarians of the first batch separately, with hourly observations, *D. bengalensis* was shown to consume *An. gambiae* s.l. larvae, though their predation rates decrease with time [95]. Turbellarians play an important role as predators in ephemeral ponds because their eggs can survive dry periods [93]. Planarians are therefore an excellent candidate for inclusion in malaria biocontrol programmes.

Therefore, there is sufficient evidence from field surveys backed by feeding experiments that several taxa of aquatic macroinvertebrate predators can potentially be used as biocontrol agents against malaria vectors.

### Mosquito population control trials

The assessment of a predator’s larval predation efficacy in the field is a critical step toward its use in biocontrol. Several large-scale experimental studies have been carried out to assess whether aquatic macroinvertebrate predators could be used for future malaria vector biocontrol.

As discussed above, the Odonata comprises several promising taxa for use as biocontrol agents for reducing malaria vector populations. This is because their nymph and adult life stages feed voraciously on larvae and adult mosquitoes. For instance, dragonflies and damselflies feed on both larvae and airborne adult mosquitoes [96]. A field study conducted at the Legon campus in Ghana revealed that an odonatan species, *Bradinopyga strachani*, can colonize concrete open containers of 120 × 60 × 40 cm^3^, and their presence decreases *Culex* and *Aedes* mosquito populations [71]. A successful project in Panama used indigenous dragonflies and damselfly naiads in water-filled tree holes to reduce the number of *Aedes*, *Anopheles*, and *Culex* species [97]. In Myanmar, a 2–6 month period of augmentative release of predatory larvae of a dragonfly, *Crocothemis servilia*, suppressed the larval and adult populations of *Ae. aegypti* by 96% [98]. After 15 days in semi-field conditions, five coexisting odonate species, *Aeshna flavifrons*, *Coenagrion kashmirum*, *Ischnura forcipata*, *Rhinocypha ignipennis*, and *Sympetrum durum*, significantly reduced *Cx. quinquefasciatus* larval densities in India, and when the odonate nymphs were removed after 15 days, the mosquito larval density rebounded significantly [99]. A pilot field study conducted in Maine, northern USA, used readily available predatory larvae of a dragonfly, *Cwcothemis servilia*, and revealed that this species could suppress *Aedes aegypti* populations to a very low level during the rainy season [98]. In the northern USA, dragonfly nymphs can be purchased for biological control and they are typically supplied from Massachusetts and North Carolina [100]. Similar attempts are yet to be conducted in Africa, where malaria is endemic despite the artificial rearing of these predators and releasing in mosquito larval breeding sites being considered an appropriate measure for mosquito population reduction [96].

Copepods, for example, are promising biocontrol agents for mosquito population reduction due to their high reproductive rate [90]. Copepods such as *Mesocyclops thermocyclopoides* significantly reduce *Aedes*, *Anopheles*, and *Culex* larval populations [90]. In the case of *Aedes* mosquitos, there is systematic evidence for the effectiveness of copepods in dengue vector control [101]. The introduction of *Mesocyclops woutersi*, *M. thermocyclopoides*, and *M. pehpeiensis* into all wells, cement water storage tanks, and ceramic jars with average capacities of 2700 and 27 L, respectively, in Phanboi, Vietnam for 12 months resulted in a 97% decrease in *Aedes aegypti* population [89]. The predatory efficacy of the copepod species *Mesocyclops longisetus* collected in and around Erode, Tamil Nadu, India, was investigated in laboratory and field studies against the malarial vector *An. culicifacies*, and their effective predation on first-instars and second-instars were 47% and 36%, respectively, compared with 3% and 1% on third- and fourth-instars, respectively [102]

Notostracan tadpole shrimp is another macroinvertebrate predator taxon with a biocontrol potential against *An. gambiae* s.l. because they are adapted to ephemeral aquatic habitats and rice paddy fields, the *An. gambiae* s.l.’s preferred breeding habitats [59, 103]. Between 1957 and 1959, the reared and released crustacean species *Triops granaries* reduced the population of *An. gambiae* s.l. larvae in temporary breeding habitats around huts in Mirrich village, Somalia [59].

*Toxorhynchites* mosquito larvae are predators of other mosquito larvae with whom they share a habitat [104]. While this genus is found throughout the tropics, its efficacy as a biocontrol agent against malaria vectors *An. gambiae* s.l. in Africa remains to be tested. *Aedes aegypti* larval populations were suppressed by predatory *Toxorhynchites moctezuma* mosquito larvae released systematically in the Caribbean Island of Saint Vincent and the Grenadines. After 5 months of sustained predator release, all the *Ae. aegypti* indices were lower in the treated village than in the untreated villages [105].

All of these suggest that, in addition to the currently known control interventions, aquatic macroinvertebrate predators could be used as a supplementary biological control tool to reduce malaria vector populations. However, data on larger field trials using aquatic macroinvertebrate predators as biological control tools against *An. gambiae* s.l. and other important vector species are scarce probably due to limited funding, yet larger interventions would bring important information about scalability. Field experiments are urgently needed to confirm the potential of aquatic macroinvertebrates as a biological tool in the control of malaria vectors at a larger scale.

### Using predator kairomones to deter ovipositing *An. gambiae* s.l.

Kairomones are “allelochemicals produced, acquired, or released as a result of an organism’s activities that, when it comes into contact with an individual of another species in the natural environment elicits in the receiver a behavioral or physiological reaction that is adaptively beneficial to the receiver but not to the emitter” [106]. A large portion of an insect’s behavior is mediated by information derived from chemicals in its environment, which can act as attractants or repellents [107]. Kairomones, for example, play a role in mosquito attraction and sexual partner selection, as well as mediating communication within and between insect taxa [108, 109].

Knowledge of insect behavior can be used to develop long-term control strategies for disease vectors. For example, the exploitation of a kairomonal attraction to host odors resulted in the development of traps that use info-chemicals such as carbon dioxide (CO_2_), lactic acid, ammonia, carboxylic acids, and 1-Octen-3-ol to trap host-seeking insects [108–110]. Thus, it has been demonstrated that *An. gambiae* s.l. and *An. funestus* are highly attracted to traps baited with host-derived semiochemicals [10].

Several aquatic macroinvertebrate predator taxa share *An. gambiae* s.l.’s larval habitats [45, 66]. The predators shed kairomones in the aquatic larval habitats, which can elicit a variety of behavioral responses from mosquitoes, including female mosquito oviposition site avoidance, and also negatively affect the life history traits of the offspring, such as delayed larval development, reduced body size, and survival of the emerged adults [110–112]. A laboratory study in Kenya has demonstrated that *An. gambiae* s.s. lay fewer eggs in water conditioned with backswimmers *Notonecta* sp. compared with unconditioned water [6]. In a rice field experiment, *An. gambiae* s.l. larvae were not commonly seen in borrow-pits inhabited by backswimmers (Notonectidae) [93]. Similarly, backswimmer *Notonecta maculata* repelled ovipositing *Culiseta longiareolata* and *Culiseta longiareo* females in outdoor and laboratory experiments with *An. gambiae* s.s., respectively [6, 111, 113]. Thus, different mosquito species may respond similarly to deterring kairomones from similar predator taxa potentially to avoid predation risk on their offspring. Differences in larval avoidance responses to kairomones produced by larval predation are also thought to be the cause of habitat divergence between *An. gambiae* s.s. and *An. coluzzii* [40, 41].

Chemical cues undoubtedly play an important role in selecting oviposition sites, and when used properly, may provide promising results in mosquito population control. Currently, well-known kairomones mediating female mosquito oviposition behavior that have been used as an attractant baited with insecticides to control malaria vector population come primarily from aquatic plants, algae, and bacteria [101, 114]. Despite the enormous laboratory and field studies that demonstrate the role of aquatic macroinvertebrate kairomones in repelling gravid female mosquitoes from oviposition sites, very little is still known on the chemical compound profile emitted by many promising predators, e.g., backswimmer *Notonecta* sp., diving beetles, and dragonfly nymphs, limiting the use of their kairomones as a vector control tool against malaria vectors. The predator kairomones can be used as a malaria vector control tool in a push (deterrence)–pull (insecticide-baited attractant) approach [115, 116]. The discovery of attractant and repellent compounds derived from aquatic macroinvertebrate predators that could potentially be used for malaria vector control would open completely novel new research avenues. As a result, future research should concentrate on identifying specific volatile compounds released by macroinvertebrate predators that have the potential to be used in a push–pull strategy to control malaria vector populations [117].

### Bottlenecks limiting the use of aquatic macroinvertebrate predators as biocontrol tool

#### Knowledge gaps in ecology

Biocontrol by macroinvertebrate predators is likely to be the most cost-effective when *An. gambiae* s.l. larval breeding sites are present, and larvae are developing in some numbers. Planning effective interventions therefore requires gathering important basic knowledge on rainfall seasonality, the resulting distribution of aquatic habitats, and the extent of overlaps between aquatic macroinvertebrate predators, the targeted prey species, and alternate food sources. Baseline longitudinal studies that include the identification of areas with high densities of water bodies and sampling of mosquito larvae, among other prey, are therefore crucial to the implementation of augmentative release of predators in target habitats. Aquatic macroinvertebrate predators tend to be more common in more stable and larger water bodies, but different taxa vary in their preferred habitats [5, 118]. Furthermore, *An. gambiae* s.l. larvae were shown to be particularly abundant in small temporary water pools where fewer aquatic predators are present [5, 119]. Targeting this species would therefore require using adult predators actively searching smaller temporal larval habitats for prey, or developing a method for effectively detecting smaller larval habitats, or treatment with the egg or larval stages of predators. In addition, creating stepping stones from which these predators can colonize or reach these temporal water bodies would also be beneficial. Either of these approaches requires gaining an in-depth knowledge of the local ecology and dynamics of both prey species and the predators to be considered for biocontrol. At present this knowledge is far too sparse to envisage the modeling exercises required for planning interventions effectively and estimating their likely impact on transmission.

### Rearing and production challenges

Difficulties associated with rearing aquatic macroinvertebrate predators under captivity have been cited as one of the limiting factors for their adoption as a biocontrol tool against malaria vectors. Rearing aquatic predators is typically complicated by the fact that many species readily cannibalize conspecifics when given the opportunity, and this constrains the design of rearing procedures [120, 121]. Another level of complexity stems from their often complex holometabolous life [122, 123]. For instance, the long and complex lifecycle of the dragonflies (8–17 larval instars depending on species) makes them both difficult to rear, and, in the field, slows down their population response to malaria vector dynamics [124–126]. Some species, such as the Asian dragonfly *Bradinopyga geminata*, could not be reared in captivity to adulthood [98, 127, 128]. In America, an attempt to rear the backswimmer *Buenoa scimitra* for controlling *Cx. quinquefasciatus* partially failed due to difficulties in establishing similar natural field conditions in the rearing facilities [129]. However, another study reported that the eggs from that species could be stored for 263 days, and newly hatched individuals successfully grew to the adult stage, thereby enabling mass-rearing for the control of *Cx. quinquefasciatus* [130]. This example highlights the fact that many rearing and production challenges could be overcome provided enough resources in applied research are available. Despite the slowdown in chemical control efficacy, the focus on, and funding of, biocontrol research remains limited. In most cases, mass-rearing of macroinvertebrate predators is attempted with limited resources on the back of small projects and budgets. Therefore, the scope for trial and error and technological investments is similarly limited and thus the current understanding of the conditions influencing predator quality and performance in the field remains [131]. More resources are therefore needed to conduct the semi-field and field experiments needed to establish and optimize the rearing methodologies for the most promising aquatic macroinvertebrate predator taxa. It is paramount for these predators to be evaluated as biocontrol agents in larger trials, leading to their potential approval as additional tools against malaria vectors.

### Challenges in integration with chemical vector control

Depending on its scale and intensity of implementation, biocontrol of larval stages of malaria vectors through natural predators could potentially be a cheap and simple approach to implement [3]. The local rearing and distribution of predators would perfectly fit the current agendas of sustainability and community-based approaches, as these would establish local knowledge-based skills and jobs, and result in intervention that can usefully complement the World Health Organization (WHO)-recommended governmental-run ITN distributions and IRS spraying programs. However, the effective integration of macroinvertebrate predators with such core vector control as an intervention raises several practical questions. For instance, all insecticide classes, including the pyrethroid class used in mosquito control, are toxic to all insects and could potentially affect the adult stages of Ephemeroptera, Trichoptera, Hemiptera, Plecoptera, and Coleoptera used in biocontrol [132–135]. This is compounded by the fact that while mosquitoes develop stronger insecticide resistance in response to more powerful vector control formulations, the susceptibility of insect predators’ populations may remain high. Thus, currently used pesticides may reduce natural mosquito predator populations more effectively than target mosquitoes, and predators may die out over time [136]. Another issue stems from the leaking of pesticides used for vector control into rain water and aquatic habitats [137]. This and other sources of pesticides and contaminants associated with human activities associated with agriculture (pest control, herbicide, and fertilizing) may render aquatic habitats unsuited for aquatic predators. Thus, the loss of aquatic diversity associated with various human activities negatively affects natural mosquito larvae predator populations and could also constrain the use of aquatic macroinvertebrate predators as a biocontrol tool for malaria vector population suppression. An altenative approach would consist of using *Bti* instead of chemicals in integrated approaches using macroinvertebrate predators. However, to inform the design of a sustained integrated malaria vector control approach, it is necessary to investigate the effect of other vector control tools and other aquatic pollutant-generating activities on macroinvertebrate predators.

## Conclusions

Since the artificial mass-rearing of the aquatic macroinvertebrate predators and deployment to aquatic mosquito larval breeding habitats could be an ideal approach, we envisage that this may be a short term-solution constrained by insufficient labor and funds. Most of the aquatic macroinvertebrate predators of mosquitoes thrive in larger aquatic water bodies with short grasses for egg-laying and refuge during vulnerable stages from other predators. Highly effective aquatic macroinvertebrate predators of mosquitoes such as dragonflies, predacious diving beetles, and damselflies can fly across aquatic larval habitats to search for prey. We recommend that the naturally occurring water bodies with grasses that naturally sustain high populations of these should be conserved to allow for multiplication and reproduction of these predators for long-term suppression of mosquito population in addition to other control strategies.

## Data Availability

Not applicable.

## Abbreviations

DDT: Dichlorodiphenyltrichloroethane
WHO: World Health Organization
RBM: Roll Back Malaria
IRS: Indoor residual spraying
ITNs: Insecticide-treated nets
DNA: Deoxyribonucleic acid
(rDNA): Ribosomal deoxyribonucleic acid
PCR: Polymerase chain reactions
